# Deformation of the Durom Acetabular Component and Its Impact on Tribology in a Cadaveric Model—A Simulator Study

**DOI:** 10.1371/journal.pone.0045786

**Published:** 2012-10-29

**Authors:** Feng Liu, Zhefeng Chen, Yanqing Gu, Qing Wang, Weiding Cui, Weimin Fan

**Affiliations:** Department of Orthopedics, the First Affiliated Hospital with Nanjing Medical University, Nanjing, China; University of Akron, United States of America

## Abstract

**Background:**

Recent studies have shown that the acetabular component frequently becomes deformed during press-fit insertion. The aim of this study was to explore the deformation of the Durom cup after implantation and to clarify the impact of deformation on wear and ion release of the Durom large head metal-on-metal (MOM) total hips in simulators.

**Methods:**

Six Durom cups impacted into reamed acetabula of fresh cadavers were used as the experimental group and another 6 size-paired intact Durom cups constituted the control group. All 12 Durom MOM total hips were put through a 3 million cycle (MC) wear test in simulators.

**Results:**

The 6 cups in the experimental group were all deformed, with a mean deformation of 41.78±8.86 µm. The average volumetric wear rate in the experimental group and in the control group in the first million cycle was 6.65±0.29 mm^3^/MC and 0.89±0.04 mm^3^/MC (t = 48.43, p = 0.000). The ion levels of Cr and Co in the experimental group were also higher than those in the control group before 2.0 MC. However there was no difference in the ion levels between 2.0 and 3.0 MC.

**Conclusions:**

This finding implies that the non-modular acetabular component of Durom total hip prosthesis is likely to become deformed during press-fit insertion, and that the deformation will result in increased volumetric wear and increased ion release.

**Clinical Relevance:**

This study was determined to explore the deformation of the Durom cup after implantation and to clarify the impact of deformation on wear and ion release of the prosthesis. Deformation of the cup after implantation increases the wear of MOM bearings and the resulting ion levels. The clinical use of the Durom large head prosthesis should be with great care.

## Introduction

Large-head metal-on-metal total hip prosthesis designs with non-modular acetabular components theoretically increase range of motion, reduce wear, and decrease dislocation rates [1], [2]. Early and mid-term reports have produced encouraging results with some non-modular large-head MOM total hip acetabular designs [3]. The longest-term results of follow-up data with these designs to date are approximately 8 to 10 years and are available for only one design (Birmingham Hip Resurfacing, Smith and Nephew) [4]. Few or no long-term data are available for the majority of non-modular large-head MOM total hip devices currently in use.

Unacceptably high revision rates of the non-modular large head MOM hip replacements have been reported recently [5], [6]. The biological effects of wear products generated by MOM components made of CoCrMo alloy also remain a major cause of concern. The term of ARMD (Adverse Reaction to Metal Debris) has been coined to describe the inflammatory reactions around joints, including large joint effusions, pseudotumour formation and aseptic soft tissue necrosis resulting from these reactions [7], [8]. The systemic toxicity of cobalt and chromium ions on the vital organs and the immune system is also of significant concern [9]. Although the relationship between clinical performance and ion levels is still unclear [10], some researchers consider that malfunction and unexplainable groin pain may be correlated with high levels of metal ions [11], [12]. Some surgeons have abandoned the use of large-head metal-on-metal total hip replacements for the above reasons [13].

Multiple variables influence serum metal ion levels after MOM total hip arthroplasty. Tribologic factors, such as increased sphericity, increased carbon content, decreased clearance and surface roughness, have been shown to decrease wear and therefore, presumably, serum metal ion levels [14], [15]. Excessive abduction and anteversion of the acetabular cups might also be a risk factor for elevated metal ion release [16].

In order to preserve more bone stock and to achieve fluid-film lubrication, non-modular MOM shells for resurfacing or total hip arthroplasty have been manufactured with thinner walls to maximize femoral head sizes. Initial stability of the acetabular components can be achieved with press-fit fixation. However, despite the potential benefits of this approach, deformation may occur around the peripheral rim of the cup. This deformation may damage the fluid-film lubrication between the cup and the femoral head, increasing the wear rate and releasing more metal ions [17], [18], [19]. The main objective of this study was to observe and detect deformation of the Durom total hip prosthesis's cups inserted into fresh cadaveric acetabula, thus to clarify the relationship between deformation, volumetric wear rate and ion levels using a simulator test.

## Materials and Methods

### 1. Durom cup

The Durom large head total hip prosthesis cup (original design) is a non-modular acetabular component and uses a high-carbon, forged chromium-cobalt bearing surface with a titanium plasma spray surface for bone ingrowth. The implant is a 165° truncated hemisphere with an elliptical shape providing a 2-mm press fit circumferentially. Initial stability is achieved by the press-fit technique at the rim and the purchase of the peripheral rim fins in the acetabular bone.

### 2. Cadaver pelvis and preparation of the acetabulum

All procedures were reviewed and approved by Ethics Committee of the First Affiliated Hospital with Nanjing Medical University. Three fresh adult cadaver pelves (provided by Nanjing Medical University) were harvested and were cleaned of soft tissue, preserving the sacrospinous and sacrotuberous ligaments. The specimens were wrapped in saline-soaked gauze, covered with plastic, and stored at −20°C until the insertion of the Durom cups. On the day of testing, the specimens were thawed for 12 hours at room temperature. Detailed demographics of the donors are presented in Table 1. Bilateral acetabular replacements in all cadaver pelves were performed by a senior surgeon (Feng Liu) who inserted the Durom cups.

**Table 1:** Demographics of cadaver hip donors.

Specimen	Gender	Age(y)	Height(cm)	Weight(kg)	BMD(g/cm^2^)	T-score
1R(right hip)	M	49	173	73	1.061	0.8
1L(left hip)					1.039	0.7
2R(right hip)	M	52	170	68	1.302	1.0
2L(left hip)					1.381	1.2
3R(right hip)	M	47	174	71	1.012	0.4
3L(left hip)					1.005	0.2

With the femoral heads removed from the specimens, the labrum and teres ligaments were excised. After all of the cartilage had been removed and the reamer neared the base of the notch, reaming was directed at an angle of 45° inclination and 15° to 20° anteversion. Larger reamers were used sequentially until the anterior and posterior walls were firmly engaged. Then Durom cups of the same size as the last reamer were fitted under 3D digital image correlation detection (3D DIC).

### 3. 3D Digital Image Correlation (3D DIC)

**Figure 1:**
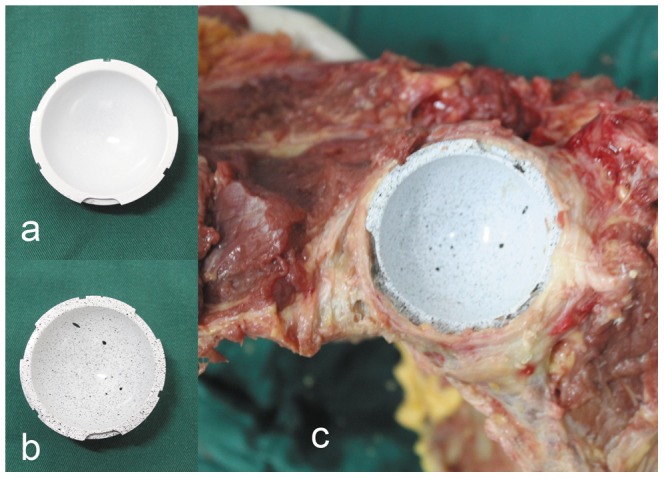
(a)Durom cup after white sprayed (b)Durom after black dots sprayed (c) Implantation of the Durom cup.

After impaction, the deformation of each cup was assessed by 3D digital image correlation (VIC-3D Correlation Solution, Inc. SC, USA, Precision: 1 µm). 3D DIC is a computer vision technique which is used to track the surface displacements of deforming materials. Before testing, proper preparation of the specimens is required. Black and white dots on the material surface are an absolute requirement for measuring the deformation. We initially sprayed a thin layer of white paint and then used a black spray to make black dots on the inner surface of all the 12 Durom cups (Figures 1a, 1b).

Before the 3D DIC measurement, hand-made support bases were made from plastic foam according to the size of the metal cups. After being put gently inside the foam support base, the foam supports with the cups were fixed on the base of the VIC-3D firmly. 6 Durom cups scheduled for implantation were then fixed for DIC detection, and the primary images were obtained. After that, the cups were removed from the support bases and impacted into the bone acetabula with 45°inclination and 15° to 20° anteversion. After the Durom cups were set in position, it was important to ensure that the cups were firmly engaged by the anterior and posterior walls, and that the rim fins of the cups had a firm purchase on the peripheral acetabular bone (Figure 1c). Then 6 specimens were inspected under 3D DIC again to detect any possible deformation. Twenty-four hours later, the specimens were examined again, and then the cups were gently removed from the bone acetabula. On the 7^th^ day, the third examination was conducted.

### 4. Simulator testing

**Figure 2:**
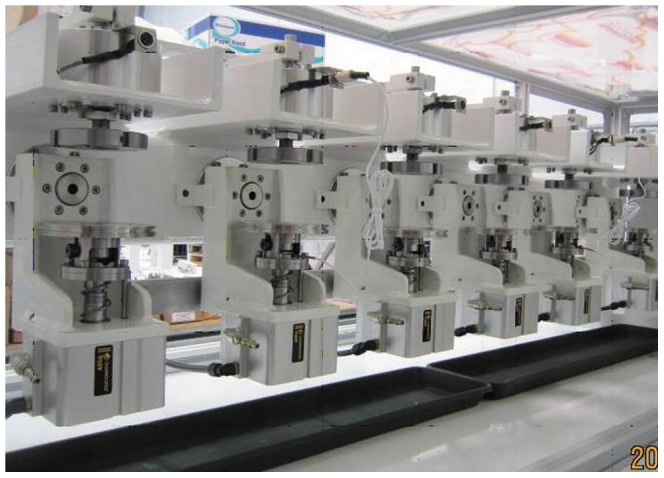
Six-Station Prosim 2 Hip Simulator (Simsol, Stockport, UK).

After the deformation detection test, all the painted cups were ultrasonicated in 95% ethanol for 2 hours, and then the paint was removed by running water avoiding the damage to the inner surface of the cups. The six Durom cups which underwent deformation testing constituted the experimental group. The remaining 6 size-paired intact cups were the control group. They were tested in six station Prosim 2 hip simulators (Simsol, Stockport, UK) (Figure 2). The total of 12 cups were randomly divided into the two groups and housed randomly in different stations. The acetabular components were housed in stainless steel fixtures, supported by polymethyl methacrylate (PMMA) cement that was cast against the back ensuring full support. Flats were machined on the reverse of the cups to prevent rotation of the cups within the cement mantle and to ensure accurate repositioning of the components following each measurement.

**Figure 3:**
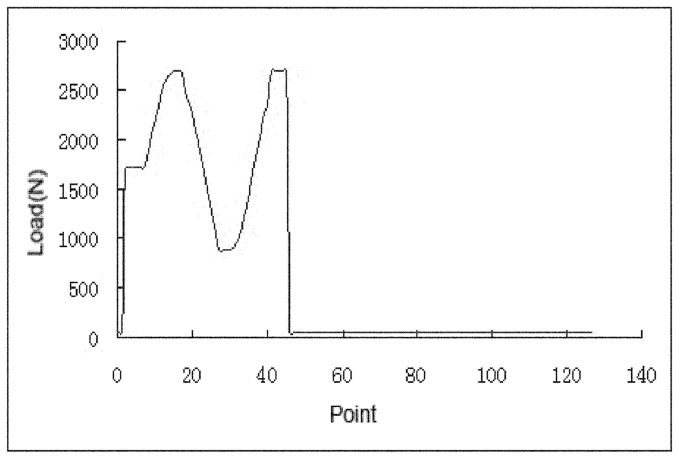
The loads used in hip simulator study.

The acetabular cups were mounted at 45° inclination and 15° anteversion and the rotational position were identical to their positions in acetabula. The femoral components were positioned vertically below the cups resulting in the contact region developing on the pole. The simulator had a flexion–extension range of −15° to 30° (on the head) and internal–external rotation of ±10° (on the cup). The motions were 90° out of phase to generate open elliptical and “figure of 8” wear paths to produce clinically relevant amounts of cross shear. Twin peak time-dependent loading was applied vertically to the femoral head. A peak load of 3 kN and a swing phase load of 280 N (ISO14242-1) were used. The test was conducted for 3.0 MC at a frequency of 1 Hz (Figure 3).

Testing was conducted in 25% (v/v) newborn bovine serum, containing 0.1% (w/v) sodium azide to retard bacterial growth. Each station was self-contained within a silicon bag containing 500 mL of lubricant to prevent evaporation. In the first 1.0 MC, the lubricant was changed after 0.33, 0.66, and 1.0 MC; between 1.0 and 3.0 MC, the lubricant was changed every 0.5 MC. At each change, the lubricant was collected and stored at −20°C for further quantification of wear debris and ion levels.

After 0.33, 0.66, 1.0, 1.5, 2.0, 2.5 and 3.0 MC, the hip simulator was stopped for measurements. The wear of the samples was quantified gravimetrically. After removal from the simulator, the test specimens were washed in detergent solution to remove all visible debris. Firstly, they were ultrasonicated in detergent solution for 10 min, then rinsed in water and soaked in Virkon to ensure all bacteria were killed. Then, specimens were rinsed with deionized water and ultrasonicated for 10 min in 70% isopropanol. Finally, after being allowed to air dry, they were put in a controlled environment for 24 h before weighing.

The components were weighed using a Mettler AT201 balance (accurate to 10 µg in a controlled environment) (Mettler-Toledo Ltd., Leicester, UK). Each component was weighed five times to determine average weight. Gravimetric data were converted into cumulative volumetric wear data (density of cobalt chromium [CoCr] 8.33 g/cm^3^).

### 5. Measurement of ion levels

Inductively coupled plasma mass spectrometry was chosen to measure ion levels in the lubricant due to the great sensitivity of this method. The detection limits were 0.1 µg/L for Cr and 0.01 µg/L for Co. An aliquot of 10 mL serum lubricant was removed from each station after 0.33, 0.66, 1.0, 1.5, 2.0, 2.5 and 3 MC. The lubricant was centrifuged at 15,000× g for 15 minutes. The supernatant was digested with nitric acid, and the final sample was introduced into the instrument and compared with a background measurement of 25% (v/v) bovine serum with 0.1% (w/v) sodium azide to verify the results.

### 6. Bearing surface profile observation

The surface profile data of each bearing after 3.0 MC were acquired using an Ultrahigh Precision Surface 3D Profiler (MiaoXAM2.5X-50X, Westwood, Tucson, USA).

### 7. Wear debris analysis

Wear debris was isolated and analyzed according to the method developed by Brown *et al* [20]. Samples of serum lubricant (10 mL) were removed from each stations at every test point and centrifuged at 15,000× g for 20 min and the supernatant was removed. The pelleted samples were digested sequentially with papain, proteinase-k, lyticase and zymolyase. Following digestion, the particles were filtered onto 1.2 µm polycarbonate filters and dried for 4 hours. Filtered samples were analyzed using a scanning electron microscope (Hitachi, S-3000N, Hitachi, Ltd,Japan). Energy dispersive X-ray analysis (EDX, Finder-1000, KYKY Technology Development Ltd, Beijing, CHN) was used to confirm the elemental composition of the wear particles. The size of the particles was determined using a Zetasizer Nano-ZS90 (Malvern Instruments ZEN3590, Malvern, UK). Using Image Pro Plus (Media Cybernetics Inc., Bethesda, MD), maximum diameter measurements were taken of 150 particles per sample to generate size distributions as a function of size.

## Statistical Analysis

Measurement data are expressed as Mean ± SD (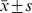). Differences between both groups were compared with Repeated Measurement Variance Analysis, Pillai's Trace and profile plots of the estimated marginal means. The p-value reported was two-sided and a value of less than 0.05 was considered statistically significant. All analyses were performed using SPSS software (Version 13.0, SPSS Inc., Chicago, IL)

## Results

### 1. Durom cup deformation

**Figure 4:**
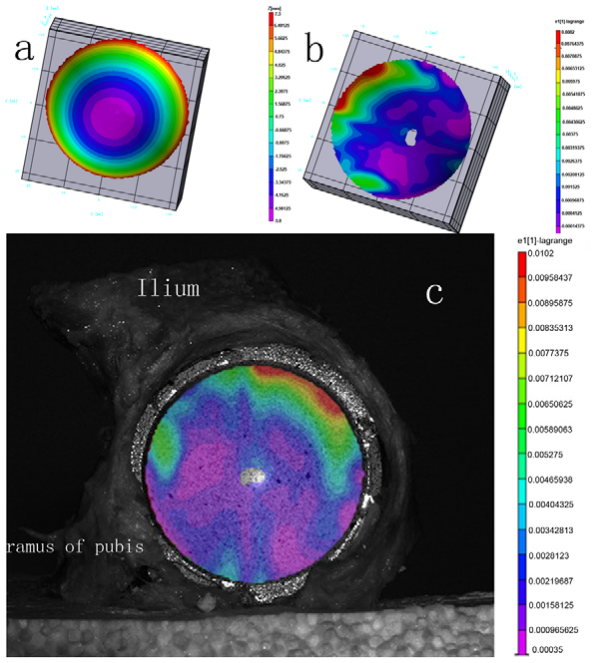
(a)The image of Durom cup before implantation (b)The image of Durom cup after implantation. (c) the image of the cup in the pelvis and location of deformation.

After the final reaming, the sizes of the six cups were 2 of 50 mm, 2 of 52 mm and 2 of 54 mm. All cups were deformed following impaction into the surgically prepared pelves (Figure 4a,b). Most of the deformations were in the posterior-superior quadrant and the area of the ramus of ischium(Figure 4c). Small areas of deformation could also be seen in other areas. The average deformation value was 41.73±8.86 µm. Detailed demographics of the deformation of each cup along with the corresponding wear value for each bearing are presented in Table 2. The deformation of cups at 24 hours and 7 days were 41.72±7.97 µm and 41.63±7.93 µm. There was no significant difference (ANOVA, F = 0.002, P = 0.998) in deformation at three testing points.

**Table 2:** Demographics of the deformation of each cup along with the corresponding wear value for each bearing.

Cup Number	Diameter(mm)	Average Deformation (µm)	Cumulative Volumetric Wear of Cup(mm^3^)	Cumulative Volumetric Wear of head (mm^3^)
1	50	43.13	4.525	8.672
2	50	30.84	4.368	8.382
3	52	32.43	4.360	8.387
4	52	48.41	4.648	8.988
5	54	46.84	4.189	8.063
6	54	48.74	4.272	8.080

### 2. Simulator testing

**Figure 5:**
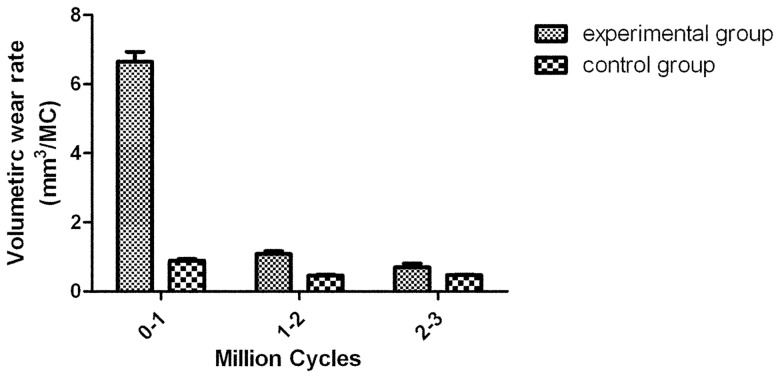
Volumetric wear rates during 0–1,1–2 and 2–3 MC.

**Figure 6:**
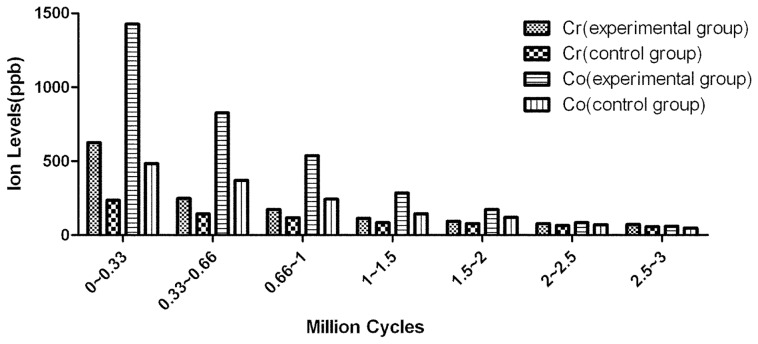
The ion levels(Levels of Co, Cr) in lubricant from 0.33, 0.66, 1.0, 1.5, 2.0. 2.5 and 3.0 MC test-points. Mean values displayed in parts per billion (ppb).

Detailed demographics of the cumulative volumetric wear of cups and heads are presented in Table 3. Two distinct phases of wear were observed for both the experimental and the control group; bedding in, which produced an elevated wear rate, and steady state, resulting in a reduced wear rate. The average volumetric wear rate in the experimental group and in the control group in the first million cycle was 6.65±0.29 mm^3^/MC and 0.89±0.04 mm^3^/MC (t = 48.43, p = 0.000). The bedding-in wear rate (0–1.0 MC) in the experimental group was almost 8 times greater than that in the control group. In the steady state (1.0–2.0 MC, 2.0–3.0 MC), the difference between the two groups became less significant. Overall, the wear rate of the experimental group was more than that in the control group over the entire testing cycle (F = 2910.916, p<0.001 and F = 1963.215, p<0.001) (Figures 5, 6).

**Table 3:** Demographics of the cumulative volumetric wear of cups and heads.

number of cycles(million)		0.33	0.66	1.0	1.5	2.0	2.5	3.0
cup (mm^3^)	control group	0.230±0.008	0.351±0.014	0.449±0.020	0.570±0.021	0.680±0.033	0.795±0.034	0.927±0.038
	experimental group	1.890±0.093	3.043±0.072	3.376±0.146	3.745±0.164	4.009±0.126	4.192±0.114	4.394±0.168
head (mm^3^)	control group	0.222±0.009	0.341±0.016	0.443±0.023	0.566±0.024	0.665±0.036	0.782±0.040	0.892±0.038
	experimental group	1.865±0.090	3.079±0.117	3.271±0.147	3.501±0.173	3.723±0.153	3.910±0.153	4.035±0.192

The levels of Co in the lubricant of the experimental group were higher than in the control group at test-points of 0.33, 0.66, 1.0, 1.5, and 2.0 MC (p = 0.000, 0.000, 0.000, 0.000, 0.011). Although there was no significant difference in Co level between the two groups at the 2.5 and 3.0 MC test-points (p = 0.102, 0.359, respectively), the overall Co levels of experiment group were higher than those of control group (F = 604.718, p<0.001).

**Figure 7:**
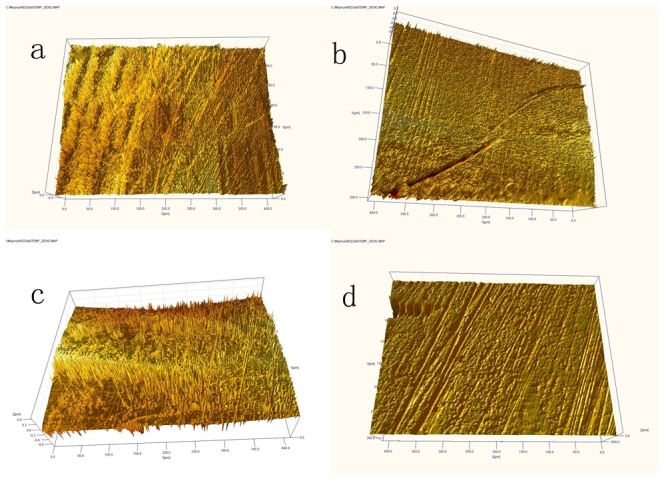
3D profile micrographs from the center of the wear zone of the femoral head (a) and cup (c) in control group after 3MC wear test. More protein deposition, shallow grooves, less wear. 3D profile micrographs from the wear zone of the femoral head (b) and cup (d) in experimental group after 3MC wear test. Less protein deposition, deeper grooves, more wear.

The levels of Cr in the lubricant of the experimental group were higher than in the control group at the test-points of 0.33, 0.66, 1.0, 1.5 and 2.0 MC (p = 0.000, 0.000, 0.000, 0.001, 0.046). Although there was no significant difference in Cr levels between the two groups at the 2.5 and 3.0 MC test-points (p = 0.094, 0.157). As the lubricant was fully replaced regularly, care must be taken when interpreting these results. The ion levels at 0.33 MC were representative of the wear between 0 and 0.33 MC, the ion levels at 0.66 MC were representative of the wear between 0.33 and 0.66 MC, and so on (Figure 7). However, Repeated measurement variance analysis indicated that the overall levels of Cr in experimental group were higher than those in control group (F = 851.469, p<0.001) (Figure 7).

### 3. Bearing surface profile observation

We observed the evident wear zone on femoral heads in deformation group. The wear zones were at the weight loading area of the femoral heads (close to the pole). On the cup side, the wear patch corresponding to the wear zone of the head was also observed. But in the control group, only mild criss-cross scratching on the contact zone was observed. The surface profile data of each cup and femoral head components' wear zone after 3.0 MC were acquired using the Ultrahigh Precision Surface 3D Profiler. Grooves were widely seen on the surface of the in both groups. The non-uniform directions of the grooves suggested the complex regimen of the wear pattern. Less protein deposition was found on the components in the experimental group, and the grooves on the wear zone in the experimental group were deeper (Figure 7).

### 4. Wear Debris Analysis

**Figure 8:**
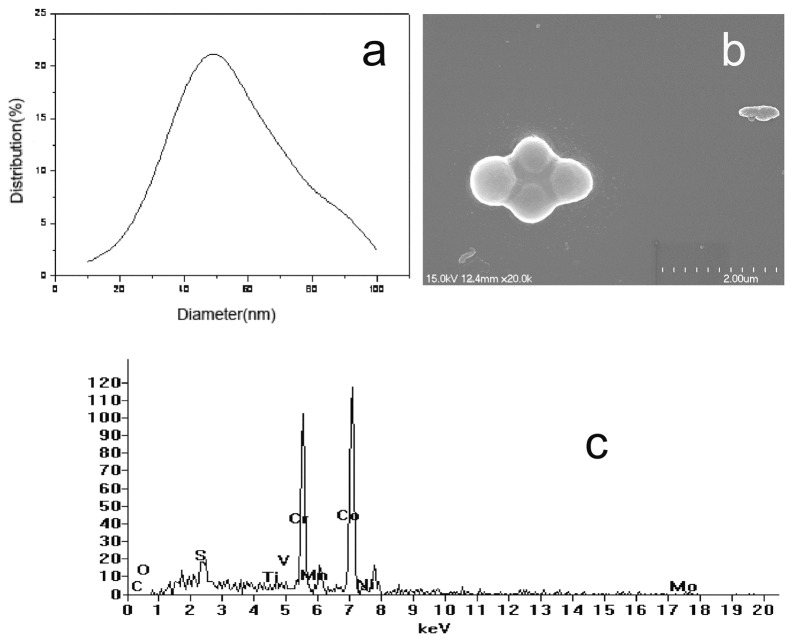
(a)Size distribution curve of wear particles (b)Image of aggregated CoCr wear particles (SEM, ×20,000) (c) The elemental composition in the wear particles by EDS analysis.

No significant differences in mean particle sizes were seen between the two groups at 1.0 MC and 3.0 MC. Predominantly aggregated round or oval globular particles with a size range of 10–100 nm were observed (mean diameter≈50 nm). EDS analysis showed that the predominant chemical components of the wear particles were cobalt and chromium (cobalt≈61%, chromium≈35%) (Figure 8).

## Discussion

Unacceptably high revision rates of the non-modular large head MOM hip replacements have been reported recently. An alternative explanation of the failure of large head metal on metal hips, both resurfacing and total hips, has been offered and is supported by evidence in the literature. With smaller and sub-optimally positioned cups, ‘edge loading’ or ‘rim-wear’ of the acetabular cup takes place. This rim-wear results in relatively large volumes of metallic wear debris which manifests itself as high ion levels in the blood of patients and a range of additional complications such as pain, swellings, pseudotumours etc. [6].

Moreover, the deformation of the acetabular component during insertion may damage the fluid-film lubrication between the cup and the femoral head, increasing the wear rate and releasing more metal ions. The Durom cup is a mono-block implant. The thickness of the cup wall is 3.7 mm and the interference of the press-fit is 2–3 mm. The Young's modulus of bone is much less than the modulus of cobalt-chrome alloy. During the implantation of the acetabular component, the bone acetabulum actually expands outwards. Kroeber M et al. [21] have confirmed this phenomenon. Theoretically, some degree of deformation would also occur on cup side during insertion more or less. In physics, F = m×a. Although there are no exact data which can describe the actual strength applies on the acetabulum and metal cup during insertion, the high acceleration accompanies with every impact of the mallet. Therefore the strength applied to the bone acetabulum by the cup is imaginably huge. On the contrary, this huge strength will also act on the cup itself. By Hooke's law, the elastic deformation ends when the material reaches its yield strength. At this point plastic deformation occurs. Our study showed that the mean deformation of the Durom cups was 41.73±8.86 µm. Many literatures also have confirmed the deformation of a variety of metal acetabular components during the insertion [22], [17], [18]. In our study, the deformations of the cups were predominantly in the superior-posterior quadrant and in areas of the ramus of ischium. One possible reason is that the bone is harder in those areas. The bone acetabula were prepared and reamed manually with hemispheric reamers, but the Durom acetabular component is an elliptically shaped, truncated hemisphere (165°) with peripheral circumferential fins, and therefore there may be some mismatch between the bone cavity and the Durom cup. This may also explain why some of the deformations appeared in other areas.

We know that 150 µm clearance of Durom bearing is a critical distance to guarantee the fluid film lubrication. In the MOM prostheses with non-deformed cups, the fluid film lubrication will be intact. But uneven (localized) 40 micrometers reduction of the clearance may cause some problems; the fluid film lubrication would change to mixed lubrication or even the boundary lubrication. Since the Durom acetabular component is an elliptically shaped, truncated hemisphere (165°), the deformation at the cup rim would lead to articular contact closer to the rim of the cup, and this edge loading effect may increase the wear rate [23], [24]. In this study, the surface of the cups and femoral heads was observed with a 3D profiler after a 3.0 MC test. Only mild criss-cross scratching on the contact zone in control group was observed which is in good agreement with Saikko's study [25] due to the similar experimental condition. On the contrary, the evident wear zone on bearing surface in deformation group was observed. These observations suggest that deformation may change the regimen of lubrication and adversely influence the wear mechanism. The decreased sphericity of bearing may result in equatorial bearing, leading to an increased frictional torque at the cup-bone interface, preventing bone in-growth, and culminating in acetabular loosening. According to our study, an average 41.78 µm deformation of the Durom cup occurred after insertion which would decrease the sphericity of the cup. The deformation of the Durom cup may possibly increase additional frictional torque at the interface.

The volumetric wear rate of the control group in the bedding-in stage was 0.8914±0.0429 mm^3^/MC which is in agreement with the results of wear rate analysis of the 55 mm ASR resurfacing joint [26]. The cumulative volumetric wear in the experimental group is 8–9 times more than that in the control group, and ion levels increased significantly. The results of this study illustrate that the deformation of the Durom cup aggravates wear and ion release.

Predominantly aggregated round or oval globular particles were observed. The particle size ranged from 10–100 nm, with a mean size of around 50 nm, in which Co accounts for about 65% and Cr accounts for about 35%, quite similar to the results of other studies [26]. This large number of small particles has the potential to distribute throughout the body via the lymphatic system, with particles found in the lymph nodes, liver, spleen and bone marrow [27]. There are also concerns about the release of metal ions. Increased level of metal ions, such as cobalt and chromium ions, in the blood and urine of patients after metal-on-metal total hip arthroplasty has been reported. Significantly increased ion levels 2–3 years postoperatively are associated with the bedding-in state of the MOM prosthesis which agreed with the results of our study. Although the volumetric wear rate of the MOM prosthesis decreases markedly after 2–3 million cycles wear; the metal ions and metal particles released will be transported and accumulated causing potential adverse biological reactions. The patients are likely to be exposed to elevated metal levels throughout the life of the prosthesis. Maezawa K et al reported that the serum chromium level did not fall and was remained after 7 years from metal on metal total hip arthroplasty in most of their patients [28]. To date, there are no scientific or epidemiologic data suggesting an increased risk of carcinogenesis or teratogenesis related to increased ions levels after the use of a MOM bearings couple [29]. However, a large number of studies have demonstrated that ions have toxic effects on the liver, kidneys and the immune system [9], [30].

To make the results more representative, this study selected specimens from medium build subjects with normal bone density as far as possible. The acetabula were reamed manually; all the procedures were carried out by one senior surgeon to minimize deviations and uncertainties. Since the deformation test is an *in vitro* test and the Durom cups may be restored to their original shape, we measured the deformation three times: the first was just after insertion; the second was 24 h later, then the cups were taken out of the specimens; and the third measurement was carried out 7 days after insertion. There was no statistical difference in deformation at three test points. Frozen cadaveric pelves were used in this study, freezing of allograft bone decreases its mechanical properties. Therefore, deformations seen in this study may be underestimated. In this study, 3D DIC was used to measure deformation, this method has obvious advantages in measuring small deformations, but there are some limitations to the use of this method. It is difficult to collect images at the very sharp edges of the Durom cups. As a result, the measured deformation data may be less than the actual deformation. We also used a digital caliper to measure deformation, but this method is too subjective to obtain accurate data. Only 6 cups were studied in this study, it is quite difficult to compare the deformation among the different size cups.

## Conclusions

Deformation of the Durom cup after implantation increases the wear of MOM bearings and the resulting ion levels.
